# Circulating microRNAs: A Potential Biomarker in TBI and PTSD War Veterans

**DOI:** 10.1177/2689288X251415246

**Published:** 2026-04-15

**Authors:** William Stewart, Christina Hejl, Rakeshwar S. Guleria, Sudhiranjan Gupta

**Affiliations:** VISN 17 Center of Excellence for Research on Returning War Veterans, Translational Science Core (Biomarkers & Neuroimaging Laboratory), Central Texas Veterans Health Care System, Waco, Texas, USA.

**Keywords:** biomarker, miRNA, PTSD, combat veterans

## Abstract

Traumatic brain injury (TBI) is a debilitating condition caused by one or more concussive insults to the head and is frequently observed in combat Veterans deployed in support of Operation Enduring Freedom (OEF) or Operation Iraqi Freedom (OIF). TBI is associated with impairment of cognitive function and development of post-traumatic stress disorder (PTSD), a psychiatric disorder. Currently, there are no validated biomarkers that can determine the detection of PTSD/TBI in circulation. In this regard, microRNAs (miRNAs) have emerged as specific and sensitive biomarkers in several central nervous system diseases and TBI. The current study evaluated the role of miRNA in circulation TBI and PTSD of OEF and OEF Veterans. While analyzed the expression profile of miRNAs in peripheral blood mononuclear cells (PBMCs) from an OEF/OIF veteran study cohort using a miRNA array and identified several miRNAs in PBMCs of TBI/PTSD compared with control subjects. We confirmed eight selective dysregulated miRNAs by independent quantitative real-time polymerase chain reaction (qRT-PCR) assays. Using bioinformatic tools, we further analyzed target gene function and enrichment analyses using Kyoto Encyclopedia of Genes and Genomes and gene ontology platforms. Based on unsupervised clustering analysis, we validated two miRNAs, miR-142-5p and miR-155-5p with their target genes like *BDNF*, *Nrg1*, and *NR3C2* by qRT-PCR analyses. Our data suggested a potential link between these two miRNAs and their target genes.

## Introduction

Traumatic brain injury (TBI) is a disabling condition affecting many veterans that have served earlier in Operation Enduring Freedom/Operation Iraqi Freedom (OEF/OIF). In OEF/OIF Veterans, TBI is primarily the result of concussive blast injuries. In addition to TBI, post-traumatic stress disorder (PTSD) is the simultaneous occurrence which is initiated after the experience of stress or a traumatic injury. It is highly comorbid condition with TBI, particularly in military populations. Therefore, military personnel who deployed in combat areas are at greater risk for both TBI and PTSD. The diagnosis of TBI primarily uses computed tomography (CT) or magnetic resonance imaging along with Glasgow Coma Scale for severity and grading of TBI.^1,2^ Although both techniques are widely used in assessing TBI, they often fail to detect lesions particularly in mild TBI due to limited sensitivity.^3^ The Glasgow Coma Scale score has also limitations in determining mild TBI in the presence of associated issues including alcohol and substance abuse, and other psychiatric disorders. Therefore, an effective biomarker for diagnosing the TBI and PTSD status is warranted.

Biomarkers have been used for the diagnosis of varieties of diseases including TBI.^4^ The U.S. Food and Drug Administration and the National Institutes of Health have also established biomarker group called BEST (Biomarkers, EndpointS, and other tools) resource for distinct roles in biomedical research and clinical practice.^5^ Currently, we have the paucity of diagnostic biomarker in detecting TBI subjects suffering with PTSD that impede the improvement of clinical evaluation and care. We, therefore, use the word TBI/PTSD in the article. In this regard, microRNAs (miRNAs) have emerged as specific and sensitive biomarkers in several central nervous system diseases and TBI.^6,7^ The miRNAs are small non-coding RNAs consisting of 21–23 nucleotides, which control the gene function through suppression of target genes.^8^ In addition to modulating the gene function, miRNAs have been observed in circulation. The association between miRNAs and various disease conditions like cancers,^9,10^ neurodegenerative diseases,^11^ neurological,^12^ TBI,^13^ psychiatric disorders^14^ in the blood have been established. The miRNAs are indicated to contribute a critical role in gene regulation in the central nervous system.^15^ However, the specific role of miRNAs in TBI/PTSD largely remains unknown. It is possible that miRNAs might contribute and be used as biomarkers in early detection or onset and progression of clinical complications following TBI/PTSD.

Recent reports suggesting immune dysfunction in TBI and PTSD war Veterans indicate the presence of cross-talk.^16–18^ Furthermore, it is also known that neuroimmune system is associated with depression.^19^ Previously, circulatory miRNAs were shown to be associated with neural activity in preclinical model of PTSD indicated a potential biomarker.^20^ However, circulatory miRNAs targeting either immune molecules or critical factors such as brain-derived natriuretic factor (BDNF) in TBI/PTSD subjects currently remain unknown. Identifying miRNA targeting these potential biomarkers may provide novel insights for the effective care and treatment of PTSD patients.

The peripheral blood mononuclear cells (PBMCs) are a diverse mixture of highly specialized immune cells that have been used as an alternative “circulating” source for biomarker analysis or analysis for active disease related alterations.^16^ Therefore, miRNA in PBMCs may reflect the molecular changes that occurred after TBI/PTSD in the brain and provide an excellent clinical setting for biomarker detection. Interestingly, these miRNAs present in the PBMCs might also play a role in immune modulation in clinically diagnosed veteran population suffering from TBI/PTSD. This will trigger the activation pattern of the immune cells finally effecting their role in inflammation and stress.

The current de-identified data were derived from the existing research data repository residing with the Central Texas Veterans Health Care System, Research & Development Committee (Protocol # 00534). The data deposited in this research data repository has examined miRNA expression in PBMCs derived from a cohort of TBI/PTSD veterans (OEF/OIF) and non-TBI/PTSD controls. On analyzing this data under an approved research protocol (2021-009), we identified small set of miRNA fingerprints from PBMCs targeting genes pertinent to TBI/PTSD and immune cells that can be used as independent biological indexes of TBI/PTSD in OEF/OIF Veterans. We further aimed to gain insight into their predicted gene targets and underlying biological pathways. We hypothesized that specific miRNAs are differentially expressed between subjects with TBI/PTSD and non-exposed healthy controls that are involved in immune modulation.

## Methods

### Participants

The project Strengthening Excellence in Research through Veteran Engagement (SERVE) is a longitudinal research program assessing post-deployment combat experiences of Iraq and Afghanistan era Veterans. U.S. military veterans, who served in support of the post-9/11 conflicts in Iraq and Afghanistan and were registered for health care at a Veterans Affairs (VA) Healthcare System in the Southwestern United States, were recruited to participate in a parent longitudinal study of factors impacting psychosocial readjustment following warzone service. This cohort is a subset of the male and female veterans OEF and OIF recruited for a parent longitudinal study that was conducted at the VISN 17 Center of Excellence for Research on Returning War Veterans, Veterans Affairs Medical Center at Waco, TX. The participants, demographic information, clinical diagnosis and TBI/PTSD measurements were published previously.^21–24^

### Sample collection and microRNA (miRNA) isolation

Peripheral blood samples (10–20 mL) were collected using BD Vacutainer (BD Biosciences, San Jose, CA, USA) by venipuncture in EDTA-coated collection tubes. PBMCs were isolated using Ficoll-Paque (GE Healthcare, Uppsala, Sweden) within 1 h of sample collection. The viability was determined by trypan-blue exclusion and were immediately frozen at −80°C until use.

Total RNA, including miRNA, were isolated from 500 μL PBMC samples of TBI/PTSD subjects and controls subjects using miRNeasy Micro Kit (Cat No./ID: 217084) according to manufacturer’s protocol (Qiagen, Valencia, CA, USA). The RNA samples were stored at −80°C until further use.

### MicroRNA-PCR array

Isolation of total RNA/miRNA from PBMC samples as described above. Next, RNA samples were used in Pathway-Focused miScript miRNA PCR Array as per manufacturer’s instruction. We have used miScript miRNA PCR Array, B-cell, and T-cell activation platform (MIHS-111Z) (Qiagen, Valencia, CA, USA). Raw data were exported from the real-time PCR instrument and fold changes were calculated using the delta Ct method (2^−ΔΔCt^). Finally, the miScript miRNA PCR array data were analyzed using Qiagen’s Data Analysis tool (https://geneglobe.qiagen.com/us/analyze/).

### qRT-PCR analysis

All qRT-PCR reactions were performed using Agilent Real-Time PCR system (Agilent, Santa Clara, CA, USA). The cDNA was synthesized using miScript II RT Kit (Cat No 218161). In brief, the total RNA was reverse transcribed into cDNA under the following conditions: 37°C for 30 min and 95°C for 5 min. The resulting cDNA was then used for miRNA expression using specific primers from Qiagen. The PCR conditions were as follows: 1 cycle of 95°C for 15 min, 40 cycles of 94°C for 15 s, 55°C for 30 s, and 70°C for 30 s min in a total of 25 µL reaction. All reactions, including no‐template negative controls, were conducted in triplicate. Relative levels of targeted miRNAs were then normalized to U6 and were calculated using the comparative Ct method (2^−ΔΔCt^). The ΔCt was calculated by subtracting the Ct values of U6 from the Ct values of the target miRNAs.

For validation of gene expression study (control *n* = 8; TBI/PTSD *n* = 12), total RNAs were extracted from the same PBMC using miRNeasy kit (Qiagen, Valencia, CA, USA). The cDNA preparation and all gene-specific primers used in the study were purchased from OriGene Technologies Inc. (Rockville, MD, USA). The cDNA preparation and PCR amplification were performed as manufacturer’s instruction. Relative levels of targeted RNAs were then normalized to GAPDH and were calculated using the comparative Ct method (2^−ΔΔCt^). Total number of samples used for the validation studies in each group is as follows:

### miRNA target computational analysis

The target genes of differentially expressed miRNAs were predicted by the following three prediction databases: TargetScan (http://www.targetscan.org), miRanda (http://www.microrna.org/microrna/home.do), and PicTar (http://pictar.mdc-berlin.de/). Specifically, we used miRNet (http://www.mirnet.ca/), which is an easy-to-use comprehensive tool integrated data from several different miRNA databases. The functional enrichment analyses were also performed in the same module, miRNet and, used both gene ontology (GO) analysis and the Kyoto Encyclopedia of Genes and Genomes (KEGG) pathway. The GO terms were identified in the biological process, cellular component, and molecular function categories. The KEGG database (http://www.genome.jp/kegg/tool/search_pathway.html) was used to map the predicted targets of the miRNAs.

### Statistical analysis

Data are presented as mean ± standard error mean (SEM). For miRNA microarrays, the differences in miRNA abundance between control and TBI/PTSD samples (background-adjusted, log2-transformed, balanced fluorescence values) as detected by microarray analysis, were calculated, and changes between the signal intensities were evaluated using Student’s *t*-tests. For the RT-qRCR, 2^−ΔΔCt^ values obtained from the RT-qPCR experiments in each group were used for the statistical analysis. Data were compared using one-way analysis of variance (ANOVA). All analysis with *p* < 0.05 is considered statistically significant.

## Results

### miRNA expression profile in the PBMCs of control and TBI/PTSD war veterans

**FIG. 1.**
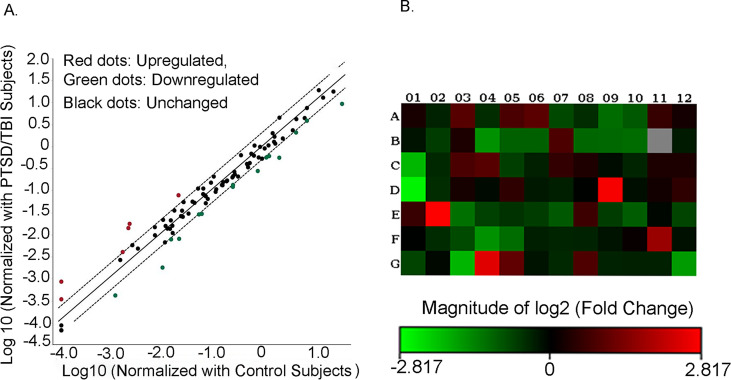
Clustergram and heatmap showing upregulated and downregulated miRNAs in TBI/PTSD participants compared with control subjects using the miScript miRNA PCR array. **(A)** Clustergram indicating the significant expression levels of miRNAs in control and TBI/PTSD participants. **(B)** Heatmap representing log2 values of 84 genes in TBI/PTSD participants group compared with control group. The color reflects the magnitude of gene expression. C_t_ values of cel‐miR‐39, miRTC, and PPC are used for calibration and RnU6‐6P is used for normalization.

Total RNA was extracted from control and TBI/PTSD groups, and RNA quality was determined. To identify dysregulated miRNAs in TBI/PTSD participants, miRNA expression profiling was carried out using miScript miRNA PCR Array for B-cell and T-cell activation pattern. This kit comprises a panel with 84 miRNAs involved in the differentiation of lymphocytes. The Clustergram and Heatmap analyses of the results from the control and TBI/PTSD group are shown in Figure 1. Three sets of data from each group were combined for significance analysis of qRT-PCR arrays. Since we used a single channel to scan the array, the data obtained from each group should be compared with the data of control group to identify differentially expressed miRNAs. We detected 11 upregulated and 14 downregulated miRNAs in TBI/PTSD participants compared to control participants (fold change >1.5) (Table 1).

**Table 1.** Fold change of dysregulated miRNAs

No	Name of the miRNAs	Fold change
1	hsa-let-7c-5p	+2.38
12	hsa-let-7e-5p	+2.25
3	hsa-let-7f-5p	+1.96
4	hsa-miR-101-3p	−1.72
5	Has-101-3p	−1.95
6	hsa-miR-126-3p	−1.71
7	hsa-miR-132-3p	−1.83
8	hsa-miR-142-5p	+2.53
9	hsa-miR-147a	−1.99
10	hsa-miR-155-5p	+1.54
11	hsa-miR-181c-5p	−1.64
12	hsa-miR-182-5p	−3.84
13	hsa-miR-184	−1.99
14	hsa-miR-195-5p	+1.56
15	hsa-miR-204-5p	−2.97
16	hsa-miR-20b-5p	+1.89
17	hsa-miR-214-3p	+2.48
18	Hsa-miR-221-3p	−2.35
19	hsa-miR-26a-5p	−2.03
20	hsa-miR-27b-3p	+1.61
21	hsa-miR-29b-3p	−3.06
22	hsa-miR-34a-5p	+2.62
23	hsa-miR-346	+5.62
24	hsa-miR-342-3p	−4.04
25	hsa-miR-99a-5p	−3.32

### Independent qPCR validation of candidate miRNA as “biomarkers”

**FIG. 2.**
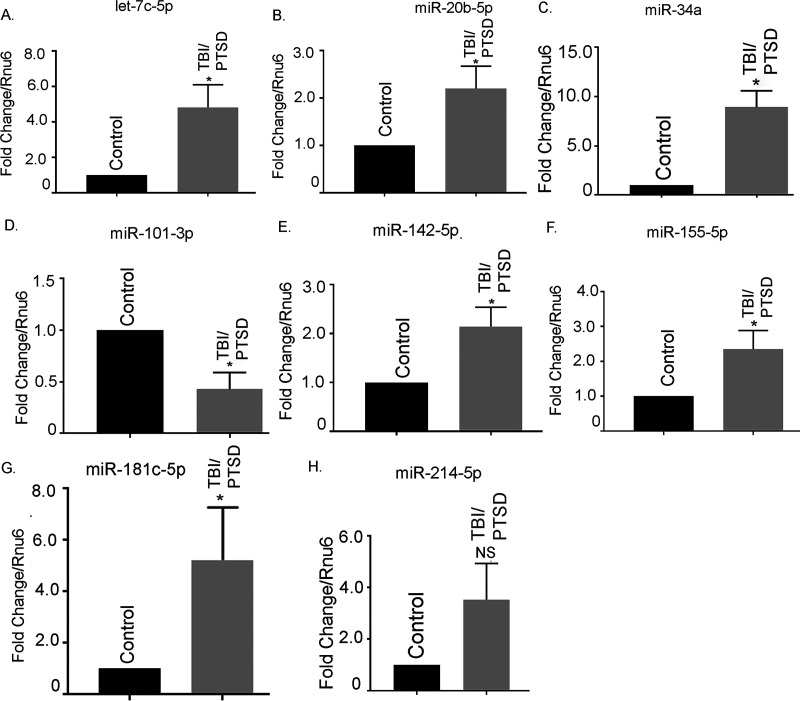
Validation of selected miRNAs in control and TBI/PTSD subjects. Using real‐time PCR, **(A)** hsa‐let7c-5p, **(B)** hsa‐miR‐20b‐5p, **(C)** hsa‐miR‐34a, **(D)** hsa-miR-101-3p, **(E)** hsa-miR-142-3p, **(F)** hsa-miR-155-5p, **(G)** hsa-miR-181c-5p, and **(H)** hsa‐miR‐214-5p were analyzed. RnU6‐was used as an internal loading control. Bar graph represents the mean ± SEM of independent experimental triplicates, *p < 0.05 compared with control subjects. NS, non-significant.

Given that TBI/PTSD is associated with PBMC miRNA modulation which eventually regulates the white blood cell function, we next examined independent qRT-PCR to assess the expression of individual candidate miRNA in PBMC specimen of control and TBI/PTSD subjects. We chose to validate the following miRNAs: let-7c-5p, miR-20b-3p, miR-34a, miR-101-3p, miR-142-5p, miR-155-5p, miR-181c-5p, and miR-214-3p in an independent cohort fashion by qRT-PCR. As compared with the controls, the TBI/PTSD cohort showed a significant increase of let-7c-5p, miR-20b-3p, miR-34a, miR-142-5p, miR-155-5p, and miR-181c-5p to 4.814 ± 1.282, 2.198 ± 0.4723, 8.95 ± 1.641, 2.349 ± 0.5368, 2.144 ± 0.3951, and 5.239 ± 2.031-fold, respectively (*p* < 0.05). The fold-change of miR-214-5p is 3.535 ± 1.399 and was non-significant. Furthermore, there was a significant decrease of miR-101-3p to 0.4288 ± 0.1632 (*p* < 0.05) in PTSD/TBI cohort compared to the control cohort. The data are shown in Figure 2A–H.

### Target gene prediction and functional enrichment analysis

To determine the biological functions of the five validated miRNAs, we first obtained their target genes from TargetScan 8.0, which provides miRNA-target genes interactions. The miRNA network analysis was performed using miRNet visual analytic platform. KEGG and GO were used to predict the signaling pathways and biological function of target miRNA. The let-7c-5p miRNA has 100 predicted conserved target genes. Several of those genes were linked with tight junction interactions, cytokine signaling, inflammation, cell cycles, immuno-modulation, and synapse. The miR-20b-3p has 4087 predicted target genes that are involved in adaptive immune system, VEGF signaling, and intrinsic apoptosis pathways. The genes are primarily involved endothelial cell proliferation, cell death, and CNS development. The miR-34a and miR-101-3p showed 754 and 956 target genes, respectively, in TargetScan 8.0. The biological function of these two miRNAs is mainly involved in inflammatory response, cell differentiation, and immune and stress response. The miR-155-5p, miR-181c-5p, and 214-5p showed 556, 1371, and 207 target genes, respectively, in TargetScan 8.0. The three miRNAs are associated with cytokine signaling, neurotrophin signaling, inflammation, immune activation, axonal guidance, gap junction trafficking regulation, cell cycle, and platelet activation. The detailed analyses are shown in Figure 3A–D and Table 2.

**FIG. 3.**
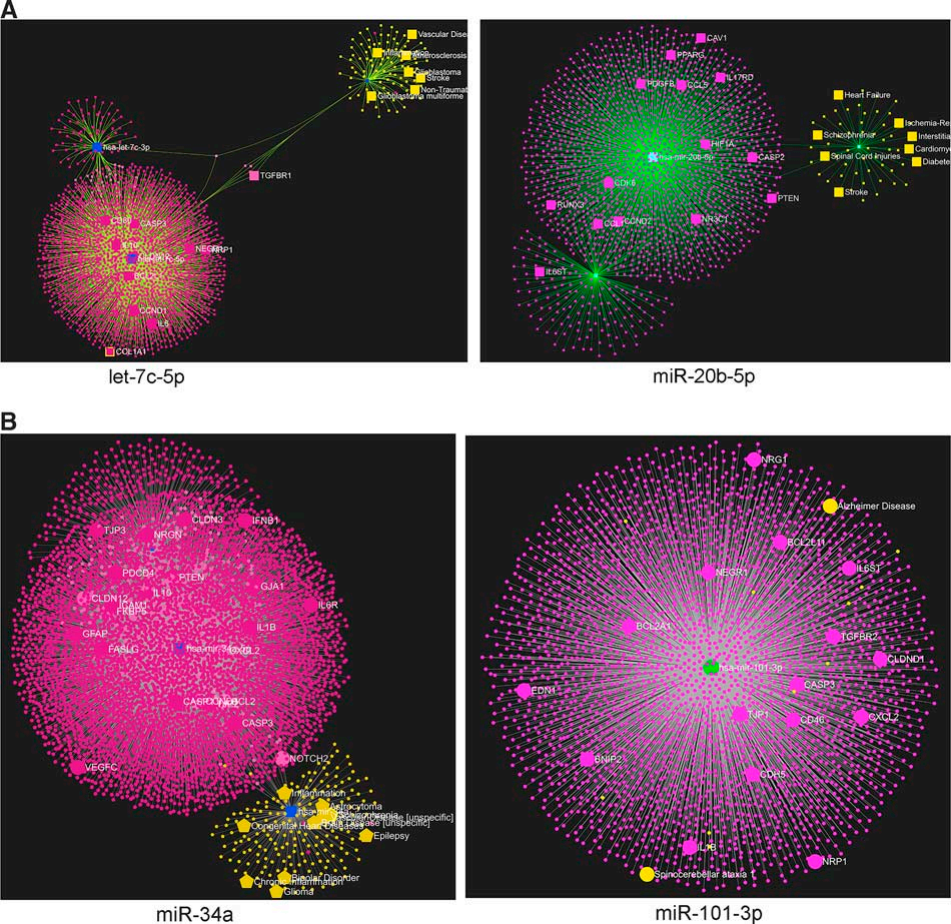
Interaction of miRNA targeting gene network using miRNet visual analytical platform. Interaction of network of genes for **(A)** let-7c-5p and hsa-miR-20b, **(B)** hsa-miR-34a and hsa-miR-101-3p, **(C)** hsa-miR-142-5p and hsa-miR-155-5p, and **(D)** hsa-miR-181c-5p and hsa-miR-214-3p. The pink color nodes depict the gene hub of the miRNA, white nodes represent potential target genes and diseases, blue node represents the miRNA, and the yellow nodes represent diseases relevant to the specific miRNA.

**FIG. 3. (Continued).**
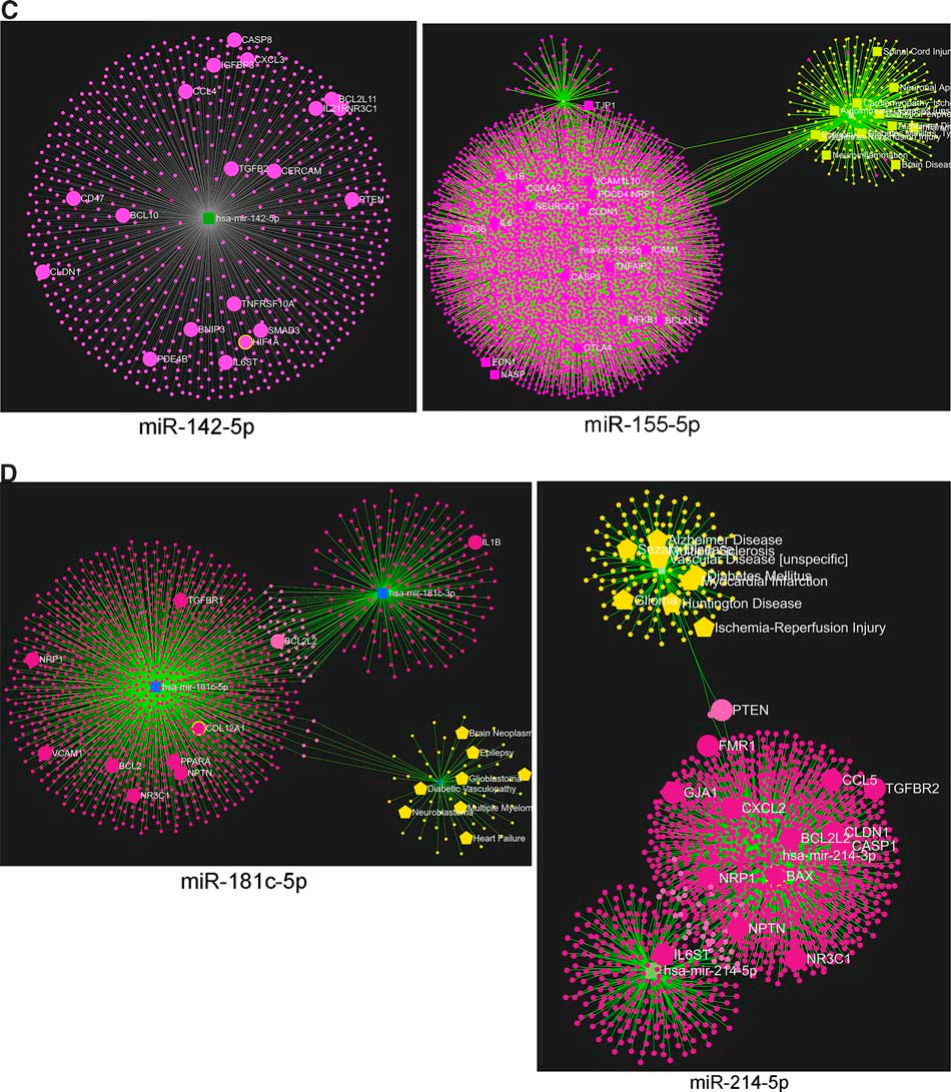


**Table 2.** Enriched Kyoto Encyclopedia of Genes and Genomes (KEGG) pathways

Sl. No	KEGG analysis: Reactome analysis	Sl. No	Gene Ontology: biological process
Let-7c-5p			
1	Cytokine-cytokine receptor interaction	1	Positive regulation of cell proliferation
2	Nerve growth factor (NGF) signaling pathway	2	Regulation of apoptotic process
3	TGF-beta signaling pathway	3	Immune system process
4	Tight junction interactions	4	Positive regulation of cell differentiation
5	Cell adhesion molecules (CAMs)	5	Regulation of neuron apoptotic process
6	Toll-like receptor signaling pathway	6	Regulation of cell differentiation
7	Intrinsic pathway for apoptosis	7	Growth
8	miRNA biogenesis	8	Positive regulation of cell migration
9	Inflammasome	9	Response to abiotic stimulus
10	MAPK signaling pathway	10	Intracellular signal transduction
11	Jak-STAT signaling pathway	11	Immune effector process
hsa-miR-34a			
1	Chemokine signaling pathway	1	Positive regulation of cell proliferation
2	Gap junction trafficking and regulation	2	Intracellular signal transduction
3	VEGF and mTOR signaling pathway	3	Response to stress
4	Tight junction interactions	4	Regulation of neurogenesis
5	Interferon signaling	5	Regulation apoptotic process
6	Cytokine signaling in immune system	6	Regulation of cell differentiation
7	Intrinsic pathway for apoptosis	7	T and B cell activation
8	Signaling by ERBB4	8	I-kappaB kinase/NF-kappaB cascade
9	Inflammasome	9	Regulation of immune system process
10	Signaling by interleukins	10	Intracellular signal transduction
11	Jak-STAT signaling pathway	11	Cell–cell adhesion
hsa-miR-101-3p			
1	Intrinsic pathway for apoptosis	1	Positive regulation of signal transduction
2	Gap junction trafficking and regulation	2	Regulation of T cell proliferation and activation
3	Interleukin-1 and -6 signaling	3	Immune system process
4	Adherens junction interactions	4	response to stress
5	Cytokine signaling in immune system	5	apoptotic mitochondrial changes
6	Innate immune system	6	Inflammatory response
7	Cytokine–cytokine receptor interaction	7	regulation of cell differentiation
8	Cell adhesion molecules (CAMs)	8	Cytokine production
9	ErbB signaling pathway	9	Regulation of protein phosphorylation
10	Signaling by NGF	10	Response to wounding
11	Jak-STAT signaling pathway	11	Regulation of apoptotic process
hsa-miR-142-5p			
1	Cytokine–cytokine receptor interaction	1	Cytokine-mediated signaling pathway
2	Tight junction trafficking and regulation	2	Regulation of apoptotic process
3	Chemokine signaling pathway	3	Central nervous system development
4	Neurotrophin signaling pathway	4	Regulation of T cell proliferation and activation
5	T cell receptor signaling pathway	5	Regulation of immune system process
6	Regulation of gene expression by HIF	6	programmed cell death
7	Interleukin-6 signaling	7	Regulation of cell differentiation
8	Signaling by GPCR	8	Generation of neurons, regulation of neurogenesis
9	Cytokine signaling in immune system	9	Brain development
10	Signaling by NGF	10	Response to wounding
11	Jak-STAT signaling pathway	11	Endothelial cell proliferation
hsa-miR-155-5p			
1	Cell adhesion molecules (CAMs)	1	Positive regulation of immune system process
2	Cytokine–cytokine receptor interaction	2	Apoptotic process
3	T cell receptor signaling pathway	3	Programmed cell death
4	Adherens junction	4	Inflammatory response
5	p53 signaling pathway	5	Regulation of cytokine production
6	TGF-beta signaling pathway	6	Regulation of cell differentiation
7	Gap junction	7	Cytokine-mediated signaling pathway
8	Axon guidance	8	Signaling by NGF
9	Neurotrophin signaling pathway	9	TRAF6-mediated induction of proinflammatory cytokines
10	Chemokine signaling pathway	10	CTLA4 inhibitory signaling
11	TGF-beta signaling pathway	11	Signaling by interleukins
hsa-miR-181c-5p			
1	Cytokine signaling in immune system	1	Cell proliferation
2	TGF-beta receptor signaling	2	Positive regulation of cell proliferation
3	Immune system	3	Regulation of response to stimulus
4	Signaling by interleukin	4	Response to extracellular stimulus
5	Generic transcription pathway	5	Cell surface receptor signaling pathway
6	Diseases of signal transduction	6	Regulation of endothelial cell proliferation
7	Signaling by Wnt	7	Protein phosphorylation
8	Toll-like receptor signaling pathway	8	Response to abiotic stimulus
9	PPAR signaling pathway	9	Endothelial cell proliferation
10	Cell adhesion molecules (CAMs)	10	Cell–cell adhesion
11	Adherens junction	11	Endothelial cell migration
hsa-miR-214-3p			
1	The NLRP3 inflammasome	1	Positive regulation of signal transduction
2	Cytokine signaling in immune system	2	T cell proliferation, T cell activation
3	Immune system	3	Immune system process
4	Extracellular matrix organization	4	Regulation of neurogenesis
5	Interleukin-6 signaling	5	Cell-cell adhesion
6	Innate immune system	6	Inflammatory response
7	Axon guidance	7	Central nervous system development
8	Generation of neurons	8	Cytokine production
9	Inflammatory response	9	Generation of neurons
10	Positive regulation of T cell proliferation	10	Response to wounding
11	Central nervous system development	11	Regulation of apoptotic process

### Target gene validation for miR-142-5p and miR-155-5p

**FIG. 4.**
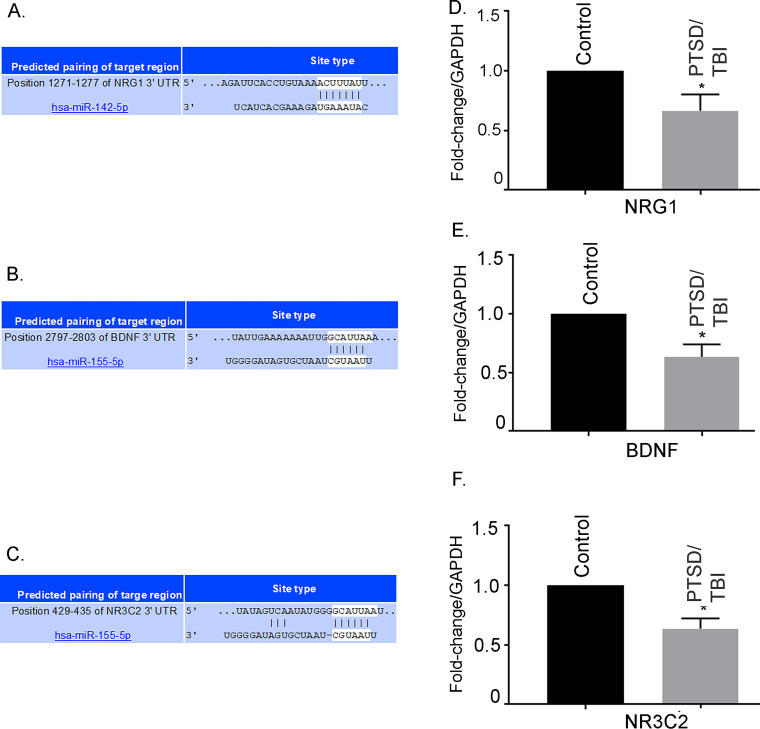
Association between target genes and miRNA expression in PBMCs. The miR-142-5p and miR-155-5p are chosen for target gene validation. **(A)** The binding site for miR-142-5p is shown in 3′-UTR of *NRG1* gene. **(B)** The binding site for miR-155-5p is shown in 3′-UTR of BDNF gene. **(C)** The binding site for miR-155-5p is shown in 3′-UTR of *NR3C2* gene. **(D–F)** The mRNA expression of *NRG1*, *BDNF*, and *NR3C2* are shown respectively. GAPDH‐was used as an internal loading control. Bar graph represents the mean ± SEM of independent experimental triplicates, *p < 0.05 compared with control subjects.

To determine the association between target genes and miRNA expression, we chose miR-142-5p and miR-155-5p for further analyses using TargetScan 8.0 and miRBase; the web-based bioinformatics tool that predicts biological target genes for miRNA. Among several predicted target genes, we found neuregulin1 (*NRG1*), *BDNF*, and *NR3C2* (a glucocorticoid receptor gene) in miR-142-5p and miR-155-5p. The miRNA: messenger RNA alignment analysis of 3′‐UTR of *NRG1* gene showed miR‐142-5p binding sites and were highly conserved among different species (Fig. 4A). The pairing of target region of 7-mer is located in1271-1277 nucleotide position of *NRG1* 3′-UTR region. Similarly, the 3′-UTR of *BDNF* and *NR3C2* genes showed miR-155-5p pairing site. The 7-mer of *BDNF* was found at 1797–2803 region and for *NR3C2* gene, the location of 7-mer was 429–435 region (Fig. 4B and C). To validate whether these genes were the target for miR-142-5p and miR-155-5p, qRT-PCR was performed using PBMCs of same cohort. Our analysis showed 0.66 ± 0.13-fold reduction of *NRG1* mRNA expression in PTSD subjects compared with control subjects (Fig. 4D). The data corroborated with increased miR-142-5p expression in TBI/PTSD subjects. A significant reduction of *BDNF* and *NR3C2* genes were observed: 0.63 ± 0.1 and 0.63 ± 0.08, respectively (Fig. 4E and F). The finding is also corroborated with upregulation of miR-155-5p in TBI/PTSD subjects as compared with the control subjects. The miR-155-5p showed target for both *BDNF* and *NR3C2* genes. Our data suggested a potential link between these two miRNAs and their target genes.

## Discission

The combat- or war-related TBI injuries are among the prevalent injuries in our military service members and prolonging in our Veteran population inducing behavioral and neurological deficits. PTSD is commonly co-morbid with TBI in OEF/OIF Veterans.^24,25^ Furthermore, PTSD is associated with mental health consequence of military personnel affecting the quality of life and enhanced morbidity risks.^26–28^ Our analysis has identified and validated seven miRNAs that are likely to represent biological indices selective for TBI/PTSD. We have also established a correlation of these miRNAs with their selective physiological pathways that may be responsible for many of the behavioral and neurological deficits. Our analysis also revealed target genes that reside in the pathways and might be responsible for mental health illness. Furthermore, the target genes for miR-142-5p and miR-155-5p were validated using bioinformatics approach. This is the first report showing an association between miRNA and targeted genes with selective physiological pathways in TBI/PTSD war Veterans.

TBI elicits two sets of injuries in the brain. The primary injury is the external force, and the secondary injury is the manifestation of a series of pathophysiological alteration in neurovascular unit including excessive inflammatory response, blood–brain barrier (BBB) breakdown and cellular apoptosis.^29,30^ Several studies have shown that miRNAs contribute a pivotal role in TBI and presumably involved in BBB damage and neuroinflammatory response.^31–33^ miRNAs are non-coding RNA molecules with 19–24 nucleotides that bind to 3′ untranslated region of mRNA, interfering the transcription machinery, diminished mRNA translation, and eventually attenuate protein level.^34,35^ PTSD is frequently associated with TBI in OEF/OIF Veterans and considered as co-morbid factor.^25,28,36,37^ Therefore, alteration of miRNAs gives us a new opportunity to understand biological underpinnings of TBI/PTSD.

Our study identified a panel of dysregulated miRNAs in TBI/PTSD subjects compared with normal subjects by qRT-PCR array. Among several dysregulated miRNAs, we validated seven miRNAs, the let-7c, miR-20b, miR-34a, miR-142-5p, miR-155-5p, miR-181c-5p, and miR-214-3p, which were upregulated in TBI/PTSD subjects. The upregulation of miRNAs appears to possess functional relevance when analyzed through TargetScan, MicroCosm, and miRBase databases. As reported, the let-7c-5p, which is a conserved and well-studied miRNA, showed potential in inflammation control and its implications in macrophage polarization.^38^ In a murine TBI model, let-7c-5p is shown to reduce neuroinflammation and promoting microglia M2 polarization, indicating therapeutic potential for neurological outcome of TBI.^39^ TargetScan analysis showed several putative targets pertinent to TBI/PTSD such as endothelin-1, nerve growth factor (*NGF*), and *CCL3*. Interestingly, study has shown that reduced *NGF* in TBI/PTSD indicated a possible link between let-7c-5p and *NGF*.^37^ Moreover, upregulation of let-7c-5p in PBMCs in our study indicates immunogenic response and may regulate the pertinent target genes during the neuropathological events. Similarly, upregulation of miR-142-5p was associated with several diseases and is regulator for organogenesis.^40^ Interestingly, our study corroborated with rat PTSD model. Both single prolonged stress and tail shock rat model of PTSD showed upregulation of miR-142-5p.^20,41^ A reduction of Npas4 gene was implicated in stress-related pathologies and miR-142-5p was the target for Npas4. Likewise, miR-155-5p showed putative target gene for *BDNF*, which is reduced in PTSD and considered as a predictor for PTSD diagnosis.^42^ Our finding of upregulation of miR-155-5p may suggest a possible reason for the reduction of *BDNF* in circulation in TBI/PTSD subjects. Regarding miR-181c-5p, several putative target genes were observed, including *CD4, NEGR1*, and *NRP1*, which tend to get affected by its upregulation. It is reported that the intensity of IFN-γ expression by the CD4^+^IFNγ^+^ cells was lower in OEF/OIF Veterans with PTSD, suggesting a possible link between upregulation of miR-181c-5p and reduction of CD4 count.^43^ Also, miR-214-3p showed several potential targets in TargetScan and miRBase including *claudin 2, claudin 5*, *NRG1*, GDNF, and neuroplastin 1. *NRG1* is involved in multiple physiological processes including neural development and synaptic plasticity.^44^ Reduced level of *NRG1* was observed in the serum of PTSD subjects, indicating a possible link between miR-214-3p and *NRG1*.^45^ Furthermore, rodent study showed neuroprotective potential of *NRG1* on BBB permeability after TBI.^46^ Together, our data suggest a link between miR-214-3p and *NRG1* in TBI/PTSD subjects. Our study also identified miR-101-5p, which was significantly reduced in TBI/PTSD cohort. Target gene analyses showed that Il1β and Il6 were targets among several others including *claudin 1, GJA1*, and *TJP1*. It is well known through clinical studies that increased circulatory inflammatory cytokines like Il6, Il1β, etc. have been observed in war Veterans.^47^ Therefore, decreased level of miR-101-5p in our sample analysis may suggest the upregulation of interleukins found in blood circulation. Together, our study identified TBI/PTSD linked miRNA signatures in PBMCs and opens new avenue for clinical applications. The identified miRNAs are critical as their target genes are related to PTSD/TBI phenotype but not necessarily the reflection of PTSD/TBI signature in Veterans. The change in certain miRNAs in PBMCs is an interesting phenomenon as these cells play a vital role in acute and as well as chronic inflammation, which is one of the main hallmarks of TBI and recently has also been considered a vital factor in cases of PTSD as well. However, our study did not confirm that these miRNAs can be directly considered as biomarkers for TBI/PTSD but as they have been identified from PBMCs, we can suggest with more confidence that these can be biomarkers for immune modulation under the clinically diagnosed cases of TBI/PTSD in our war Veterans. We also suggest that these candidates not only can be identified as biomarkers of immune modulation but if studied on a larger cohort, there is a great possibility that few of these biomarkers can turn out to be the biomarkers for TBI/PTSD Veterans population (as we have validated that some of the genes modulated by few of these identified miRNAs are also present in the brain cells that are proven to have their specific roles in TBI/PTSD). The biological correlation between the miRNA changes observed in the PBMCs and the control of the inflammatory response induced by the white blood cells (PBMCs), warrants further investigation.

Our data showed a corroboration with miRNA alteration in the PBMCs and their target genes in the TBI/PTSD cohort. Using bioinformatic tools that predict target genes, we validated the target genes for two miRNAs, miR-142-5p and miR-155-5p. Both miRNAs were reduced in TBI/PTSD cohort. The target genes that we have selected for analyses were *NRG1*, *BDNF*, and *NR3C2*. Our findings showed reduced expression of *NRG1*, *BDNF*, and *NR3C2* in TBI/PTSD subjects compared with control subjects. This is the first report that indicated a strong link between the miRNA alteration and target gene expression in the same cohort.

In summary, we report several differentially expressed miRNAs in PBMCs of TBI/PTSD subjects compared with normal subjects. These miRNAs are predicted to be associated with neuroinflammation and immune modulation in TBI/PTSD. Furthermore, alteration of miRNAs specifically, miR-155-5p and miR-142-5p, were aligned with their target gene expression, *BDNF*, *NR3C2*, and *NRG1*, provided significant association between them and predicted to be used as a biomarker candidate in TBI/PTSD veteran population. The study may provide a novel approach in identifying miRNA-footprints that can be used as biomarkers in TBI/PTSD Veteran cohort. However, the study is limited by small sample size of war Veterans. Therefore, our findings set up a stage for planning a larger cohort study to determine the role of miRNAs as predicted diagnostic and prognostic biomarkers of combat Veterans suffering from TBI/PTSD. Future prospective longitudinal studies with larger sample sizes to validate our findings and the links between miRNAs and TBI/PTSD phenotypes are warranted.

## Copyright Statement

Since all authors are employees of the U.S. Government and contributed to the article as part of official duties, the work is not subject to U.S. copyright.

## Data Availability

The data that support the findings of this study are available from the corresponding author upon reasonable request.

## Abbreviations

CNS: Central nervous system
CTLA4: Cytotoxic T-Lymphocyte Antigen 4
GDNF: Glial cell line-derived neurotrophic factor
GPCR: G protein-coupled receptors
mTOR: Mammalian target of rapamycin
PPC: Phosphoenolpyruvate carboxylase
PPAR: peroxisome proliferator–activated receptors
TGF: Transforming Growth Factor
TRAF6: TNF Receptor-Associated Factor 6
UTR: untranslated region
VEGF: Vascular endothelial growth factor
